# Battling the malaria iceberg with chloroquine in India

**DOI:** 10.1186/1475-2875-6-105

**Published:** 2007-08-07

**Authors:** Vinod P Sharma

**Affiliations:** 1 Centre for Rural Development and Technology, Indian Institute of Technology, Hauz Khas, New Delhi-110016, India

## Abstract

The National Vector Borne Disease Control Programme (NVBDCP) of the Ministry of Health, Government of India is reporting about 2 million parasite positive cases each year, although case incidence is 30-fold or more under-estimated. Forty five to fifty percent of Plasmodium infections are caused by *Plasmodium falciparum*, the killer parasite. Anti-malaria drug policy (2007) of the NVBDC recommends chloroquine (CQ) as the first line of drug for the treatment of all malarias. In a Primary Health Centre (PHC) reporting 10% or more cases of CQ resistance in *P. falciparum*, ACT blister pack is recommended and, so far, the policy has been adopted in 261 PHCs of 71 districts. The NVBDCP still depends on CQ to combat malaria and, as a result, *P. falciparum *has taken deep roots in malaria-endemic regions, causing unacceptable levels of morbidity and mortality. This policy was a subject of criticism in recent Nature and Lancet articles questioning the World Bank's decision to supply CQ to the NVBDCP. Continuation of an outdated drug in the treatment of *P. falciparum *is counterproductive in fighting drug resistant malaria and in the containment of *P. falciparum*. Switchover to Artemisinin-based Combination Therapy (ACT) in the treatment of all *P. falciparum *cases, ban on artemisinin monotherapy and effective vector control (treated nets/efficient insecticide spraying) would be a rational approach to malaria control in India.

## Background

The National Vector Borne Disease Control Programme (NVBDCP) is reporting about 2 million parasite positive cases a year, 50% of these *Plasmodium falciparum*. The WHO estimates 100 million cases in the South East Asian Region, 70% of these occur in India [1,2]. Independent studies by the Indian Council of Medical Research have unequivocally established that malaria incidence is hugely under-estimated [3-6]. Health is the state's responsibility, therefore, malaria control is carried out by the states, under the overall guidance of the NVBDCP. To monitor the impact of interventions surveillance is organized to detect malaria cases by examining fever cases in the entire country. In rural areas, blood smears are collected at fortnightly intervals by multi-purpose workers i.e. through Active Case Detection (ACD) and also collected at the Primary Health Centres (PHCs) i.e. Passive Case Detection (PCD). In urban areas, PCD is carried out at the malaria clinics. The blood smears are examined in the laboratory for parasite identification and results are used for follow-up action. Cases found positive are given radical treatment, as per the policy of the NVBDCP. This data is used in calculating epidemiological indices at the various levels of health services. PHCs reporting cases of drug failure are referred to the drug monitoring teams for further investigation on drug sensitivity in *P. falciparum*. If 25% (now reduced to 10%) of the cases tested show resistance to CQ, the drug policy is changed for the second line of drug. Thirteen NVBDCP teams routinely monitor *P. falciparum *drug sensitivity in the country. These teams are located in various regions so as to cover the entire country. *P. falciparum *monitoring for drug sensitivity is done using the World Health Organization (WHO) methodology of *in vivo *(28-day) test procedure for determining the status of resistance to CQ and other antimalarial drugs in *P. falciparum*. Malaria Drug Policy (2007) of the NVBDCP provides *inter alia *the following treatment guidelines countrywide [7].

1. All fever cases should preferably be investigated for malaria by microscopy or Rapid Detection Test (RDT).

2. The first line of treatment is chloroquine and the second line is ACT (artesunate+sulphadoxine/pyrimethamine) combination in case resistant to these formulations and to treat severe and complicated malaria, quinine will be the drug of choice.

3. Microscopically positive *P. falciparum *cases should be treated with chloroquine in therapeutic dose of 25 mg/kg body weight over three days and a single dose of primaquine 0,75 mg/kg body weight on the first day. The practice is to be followed at all levels including Voluntary Health Workers (VHWs) like Drug Distribution Centres (DDCs)/Fever Treatment Depots(FTDs)/Accredited Social Health Activist (ASHA) as well.

The antimalarial drug policy states that all *Plasmodium vivax *cases, undiagnosed fever cases, and clinical malaria cases should be treated with chloroquine in full therapeutic doses. ACT (artesunate+sulphadoxine/pyrimethamine) is the first line of antimalarial drug for treatment of *P. falciparum *in chloroquine resistant areas. Chloroquine, therefore, remains the main drug for the treatment of all malarias in India except in PHCs with 10% or more cases found resistant to it. The objective of this paper is to highlight the realistic and evidence-based malaria situation in the country, and how the changes in drug policy and efficient vector control can wipe out malaria, thus bringing out the importance of the WHO recommendation of a switch over to artemisinin-based fixed-dose combination therapy (ACT) to treat all *P. falciparum *cases (sensitive or resistant to CQ/SP).

## Discussion

Studies on re-emergence of malaria revealed countrywide presence and spread of *P. falciparum *e.g. *P. falciparum *is found in all states and union territories except Lakshadweep Island (Malaria situation 2002–2006, NVBDCP). It may be noted that *P. falciparum *occurrence is highly uneven in time and space [8,9]. Furthermore, the NVBDCP is reporting malaria epidemics in five or six states each year, and frequent focal outbreaks [10]. In some parts of the country, malaria epidemics cover two or more districts, dominated by *P. falciparum *[11-14]. Currently an epidemic of malaria is raging in Assam, a region more frequently visited by annual exacerbations [15,16]. The country-wide presence of *P. falciparum *is facilitated by inter- and intra-state population movement, particularly for civil works, agriculture, rail road construction, rural urban migration; thus providing opportunities for the mutant strains to disseminate across district and state boundaries [17-19].

**Figure 1:**
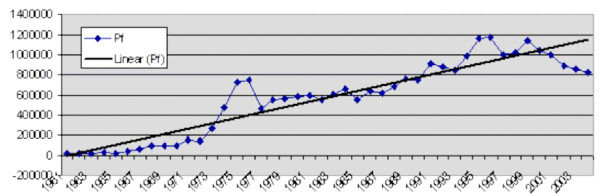
*P. falciparum *cases in India (1961–2004).

It is important to highlight the resilience of *P. falciparum *in India. During the early years of malaria resurgence [20], a focus of *P. falciparum *and detection of CQ resistant foci in Karbi-Anglong district in Assam [21] in the north-eastern states required steps for its containment and prevention of its spread to the mainland. Therefore, a special drive was launched under the *P. falciparum *Containment Programme (PfCP), financed by the Swedish International Development Agency (SIDA). PfCP was first launched in 1978 in 18 high *P. falciparum *districts in the north-eastern states, gradually extended to 55 districts (311 PHCs in 14 states) and in 1982 to 110 districts (1410 PHCs in 18 states). Despite of PfCP operations that heavily depended on Dichloro-Diphenyl-Trichloroethane (DDT) and Chloroquine (CQ), *P. falciparum *occupied larger territories and covered the entire county's transmission belts and remained firm. The purpose and the strategy of PfCP was defeated and PfCP was terminated in 1988 [22]. Epidemiological investigations revealed that *P. falciparum *was replacing *P. vivax *in central India [23]. It is noteworthy to mention that decadal *P. falciparum *rise has been substantial, an increase of 120% since the first resurgence decade (1971–80). *P. falciparum *is rising slowly but steadily [24] over these decades as shown in Figure 1.

Malaria epidemiology and its control are complicated by poverty as it is a dominant disease in poverty stricken societies. For example, Indian states with population exceeding the national average of 26.1% population below poverty line (BPL) contributed 88% *P. falciparum *in 2000 [25]. Addressing the plenary session of United Nations Conference on Human Environment at Stockholm,14 June 1972, the former Prime Minister of India Smt. Indira Gandhi said "Are not poverty and need the greatest polluters? [26] Poverty alleviation is on the national and United Nations agenda. How to fight poverty which is at the roots of all ills? Certainly malaria control is an important tool to alleviate human suffering caused by poverty and ill health. Therefore, priorities in malaria control should remain high, national and international bodies should work in tandem to eradicate this age old "King of all Diseases".

*Plasmodium falciparum *monitoring for drug sensitivity is conducted by thirteen NVBDCP teams. Monitoring is done on the line of WHO methodology of *in vivo *(28-day) standard techniques for determination of resistance in *P falciparum *to CQ and other antimalarials. India is a vast country, so it is difficult to generalize. However, between 1978 and June 2001, a total of 15,069 *P. falciparum *cases in 178 districts of 28 states and union territories have been completed. Of these 3,965 (26.3%) were sensitive to CQ; 7,661 (50.8) were S/RI; 2,142 RI resistant; 752 RII resistant (5%) and 549 (3.6%) RIII resistant to CQ [27]. In 2004, the *P. falciparum *monitoring teams collected 45,966 blood smears, of which 4,756 were positive for malaria and 3,850 for *P. falciparum *(80.95%). Results based on CQ sensitivity of 209 samples, showed Adequate Clinical and Parasitological Response (ACPR) 98 (47.9%), Early Treatment Failure (ETF) 27 (12.9%) and Late Treatment Failure (LTF) 84 (40.2%). Drug resistance to CQ is presenting countrywide, although proportion of resistant strains varies greatly [28-33]. Already the *Pfcrt *K76T mutation, an important determinant of CQ resistance, is present in >95% of *P. falciparum *isolates [34]. In addition to these clear indicators of resistance in the parasite populations, there are important changes in disease outcomes. For example, in patients with *P. falciparum *infections, acute renal and multi-organ failures have almost doubled in the last 5–7 years. Although it is not possible to attribute this directly to the decreasing efficacy of CQ, but it may be an important factor, as it has been in malaria-related deaths in African children [35]. The deteriorating trends in *P. falciparum *demands urgent radical changes in antimalarial drug policy. ACT is currently used in 261 PHCs (71 districts) as against approximately 14,000 malaria endemic PHCs. The process of adoption to ACT is painfully slow and may take a long time, until then CQ remains the first line drug in India. In 2006 a series of articles in The Lancet [36-38] and Nature [39] concerned funding of antimalaria drugs by the World Bank. The Lancet paper criticized the World Bank for funding the purchase of CQ by the Indian government. There is substantial evidence that CQ is no longer effective against *P. falciparum *in many areas of India, and under those circumstances the purchase of CQ supports a dangerous public health practice. One response to the criticism was that CQ is used in India only to combat *P. vivax*, a species against which it is mostly still effective. This statement contradicts the drug policy of the NVBDCP which recommends the use of CQ to treat all malaria cases, including *P. falciparum*, the more lethal pathogen.

It may be underscored that malaria situation in India is worsening due to ineffective vector control largely the result of DDT spraying [40] and the poor choice of antimalarials, for example India's CQ consumption in 1976 was 61 metric tons (mt) to treat 6.45 million cases (the highest since resurgence) and, in 2005, cases have been reduced by 70%, but CQ consumption has increased ten times [41]. Table 1 illustrates one example among many, of the impact of inefficient spraying and increasing dependence on CQ. In Betul district, Madhya Pradesh, a malaria epidemic was building-up due to inefficient and inadequate DDT spraying and the decreasing effectiveness of CQ. Thus, the CQ use increased enormously with no signs of epidemic abatement. Several measures were required to finally control the epidemic: a switch from DDT to synthetic pyrethroid (SP) indoor residual spraying, introduction of larvivorous Gambusia (*Gambusia affinis) *and Guppy (*Poecilia reticulata*) fishes, and on-spot diagnosis to cover all households was initiated. Then with correct diagnosis, only *P. vivax *was treated with CQ; *P. falciparum *was treated with sulfadoxine-pyrimethamine (*P. falciparum *was susceptible to it). With these changes, the district was nearly malaria free by 2005 [42].

**Table 1:** Malaria in Betul District, Madhya Pradesh, India

**Year**	**DDT (50% WP) sprayed** **in mt against 200 mt required**	**Synthetic Pyrethroid sprayed in Kg**	**CQ Tablets (150 mg base)**	**Fansidar Tablets**	**Total malaria cases**	**P. falciparum cases**
1990	Nil	Nil		Nil	496	91
1991	Nil	Nil		Nil	949	281
1992	4.00	Nil	5,00,000	Nil	805	196
1993	1.60	Nil	5,10,000	Nil	626	213
1994	4.90	Nil	5,40,000	Nil	1,503	602
1995	2.30	Nil	6,20,000	Nil	1,820	739
1996	7.40	Nil	7,70,000	Nil	2,290	662
1997	9.90	Nil	9,80,000	Nil	5,279	1,764
1998	14,90	Nil	9,60,000	Nil	8,872	3,340
1999	10.20	Nil	13,88,000	Nil	14,133	3,919
2000	18.0	Nil	20,30,000	Nil	16,764	7,126
2001	Nil	4698.4	36,83,000	49,520	18,440	7,398
2002	Nil	3512.9	25,33.000	59,094	4,911	992
2003	Nil	6429.8	29,82,000	45,400	1,080	168
2004	Nil	2528.8	16,28,000	4,600	1,063	855
2005	Nil	1352.0	12,22,000	2,740	373	193

Source: District Malaria Officer, Betul district M.P.

The WHO recommends fixed dose artemisinin combination therapy for *P. falciparum *[43]. In light of the clear evidence for CQ-resistance in *P. falciparum*, and the recommendation for using CQ as the treatment for all malaria, not just *P. vivax*, the NVBDCP should respond to the recommendation of the Lancet Viewpoint and abandon CQ, in favour of ACT. Indian states with high prevalence of *P. falciparum *have the problem of CQ resistance, although the proportion remains undetermined. CQ and sulphadoxine/pyrimethanine (SP) resistance is more pronounced in the north-eastern states and Orissa. Multidrug-resistant strains abound on the international borders with the Indian NE states [44-46]. Artesunate with SP combination (blister pack) is now recommended in drug resistant areas. In seven states in the northeastern region of the country, ACT is being introduced in one district in each state in the pilot phase. Further expansion would depend on the experience gained in the districts in north-eastern region. This switch over to ACT would take place in 71 districts in the country (261 PHCs, but there are approximately 14,000 high risk PHCs requiring ACT to treat *P. falciparum*), with provision to expand this coverage to adjacent PHCs reporting >10% CQ resistant *P. falciparum*. States have been advised to monitor the status of drug resistance in the adjacent PHCs for the expansion of ACT. PHCs lacking microscopic facility would be supplied with rapid diagnostic test kits to detect *P. falciparum*. Furthermore, artemisinin drugs are frequently used as monotherapy in the private sector, the racket of substandard and fake drugs [47], private sector working in isolation, government support to herbal drugs failing in scientific scrutiny [48] and indiscretion in the use of artemisinin are dangerous signals, and failure to address these problems may endanger life of patients and accelerate resistance. Thus the inertia may open the possibility of an epicenter of drug resistant malaria in South East Asia. Finally, withholding correct malaria treatment for unfounded reasons, lacking sound scientific basis and wise clinical judgment, is both unethical and discriminatory. Malaria is predominantly the disease of the poor lacking health equity. This inequity should be leveled by following correct drugs and drug schedule to the needy and unprotected for a relentless war against malaria. Setting the house in order is a formidable challenge. Therefore, *inter alia *policy issues must be addressed by the NVBDCP first and the foremost.
